# Impact of a virtual reality program on the cognitive and psycho-emotional state of seniors with mild cognitive impairment

**DOI:** 10.3389/fmed.2026.1872824

**Published:** 2026-07-15

**Authors:** Natalia V. Sharashkina, Olga N. Tkacheva, Elen A. Mkhitaryan, Nadezhda G. Dudchenko, Nadezhda K. Runikhina

**Affiliations:** Pirogov National Research Medical University, Russian Gerontology Research and Clinical Centre, Moscow, Russia

**Keywords:** cognitive impairment, elderly, nonpharmacological interventions, psycho-emotional health, virtual reality

## Abstract

**Background and objectives:**

Assessment of the efficacy of a virtual reality-based program in improving cognition and affect in patients with mild cognitive impairment during inpatient treatment via randomized controlled trial.

**Research design and methods:**

We compared the treatment outcomes between two groups of patients, aged 60 to 89, with mild cognitive impairment and varying comorbid conditions who underwent planned inpatient treatment at the Department of Geriatric Neurology at the Russian Gerontology Clinical Research Center. The experimental group (*n* = 31) received standard therapeutic treatment and took part in a virtual reality program for 2 weeks, while the control group (*n* = 31) received standard treatment only. All patients were assessed for cognitive function and symptoms of anxiety and depression before and after 2 weeks of treatment.

**Results:**

Both groups exhibited statistically significant improvements in cognitive function, as measured by the Montreal Cognitive Assessment scale (MoCA), and a reduction in depression symptoms, as indicated by the Geriatric Depression Scale (GDS-15). The main group also showed statistically significant improvement of anxiety and depression subscales of the Hospital Anxiety and Depression Scale (HADS), *p* = 0.007. The virtual reality program combined with standard therapy resulted in more pronounced improvement of cognitive function indicators on the MoCA scale than standard therapy alone (2.6 ± 2.1 and 1.3 ± 1.5 points improvement in group 1 and group 2 respectively), as well as a more significant reduction of depressive symptoms according to the HADS depression subscale (−1.6 ± 3.2 and −0.3 ± 1.5 points improvement in group 1 and group 2 respectively), while anxiety subscale of HADS and GDS-15 showed statistically insignificant differences, which requires discussion.

**Discussion and implications:**

Despite the obvious limitations of the presented study, such as small sample power, lack of blinding and replacement intervention in the control group, results show the promise of further research and application of Virtual reality cognitive training for the stated purposes, demonstrating the probable effectiveness as an additional nonpharmacological treatment for certain cognitive and emotional symptoms in patients with mild cognitive impairment.

## Background

Mild cognitive impairment (MCI) is a decline in cognitive abilities compared to the premorbid level that goes beyond the normative indicators, but does not deprive the person of independence and autonomy in everyday life (1, 2). MCI is often considered an intermediate stage between normal cognitive status and dementia (3, 4). Mild cognitive impairment is more common in the elderly than people in younger age groups; 15–20% of individuals aged over 60 years suffer from MCI (5). People with MCI have an increased risk of dementia, and early detection and correction of mild disorders is crucial for improving prognosis (3). According to the international data, the prevalence of MCI is 6.7% among individuals aged 60–64 years, 8.4% in the 65–69 age group, 10.1% in patients aged 70–74 years, 14.8% for those aged 75–79 years, and 25.2% for those aged 80–84 years (6). The risk of transformation into dementia within the next 2–5 years in patients with MCI is 3.3 times higher than in peers with normal cognitive functions. Depending on the cause of cognitive impairment, some patients may experience a long-term inpatient course or even improvement (7).

Numerous clinical trials have demonstrated methods for the management of mild cognitive impairment, whether it be symptomatic therapy or a strategy to slow down the progression of cognitive impairment to dementia (8). Many recent studies have shown that nonpharmacological strategies to address MCI, such as cognitive training (9, 10), specific diet supplements (12) and moderately intensive physical exercise, can be useful in improving cognitive status and managing symptoms, as well as pharmacological treatment, i.e., cholinesterase inhibitors; some of them are also promising in slowing the progression of MCI, however, the severity of the latter effect did not always have great clinical significance and varied depending on the etiopathogenetic cause of the impairment (13–17). Due to this factor, any additional treatment methods for MCI may be of value for therapy.

Nonpharmacological form of treatment can also address emotional status. In a systematic review, Chan et al. (18) analyzed 36 trials using a variety of computer-based and other methods and found statistically significant medium-to-large effect sizes in reduction of depression, an important finding as depression is common in this patient population (19).

Traditionally, cognitive training has been conducted by trained professionals in person, using paper and writing instruments, but computerized cognitive training programs are also being developed, including programs that incorporate virtual reality (VR). VR is a form of technology in which a person uses specialized electronic equipment to interact with a set of images and sounds representing a place or situation as if in a real-world environment. VR can be more or less immersive: in non-immersive types VR is conducted via standard devices like two-dimensional computer monitors with applications, while immersive types imply fully three-dimensional immersion using special sets of devices, such as glasses, headphones, headsets, tactile controllers, etc. (20). In particular, VR headsets transmit visual, auditory, and other sensations to users to make them feel as if they are in a realistic or fanciful environment. For example, in a typical task, participants might be presented with a list of grocery items to memorize, then enter a virtual supermarket to find and buy the items on the list (21, 22). VR can offer interventions in which patients interact and perform tasks in the virtual environment; this form of training can have transfer outcomes and improve visuospatial function, and thus has a key role to play in basic research and clinical practice (20).

The emergence of VR applications has led to advances in forms of healthcare including cognitive and behavioral therapy (1). Anguera et al. (1, 20) found in a study of multitasking in older adults using the custom-designed driving video game NeuroRacer that working memory training is effective in improving verbal memory and hippocampal activation in older adults (23, 24), remediating age-related deficits in cognitive control. Multitasking training in the game resulted in improved real-world multitasking ability, which was associated with middle frontal theta power in older adults (23).

A Korean randomized controlled trial by Thapa et al. (25) evaluated the effects of VR training on cognitive function and motor activity in patients with MCI. Cognitive assessments were performed by neuropsychologists according to standardized methods, including the Mini-Mental State Examination (MMSE), frontal cognitive function by trail making test (TMT) A & B, and symbol digit substitute test (SDST). The VR intervention program (3 times/week, 100 min each session) comprised four types of VR game-based content to improve attention, memory, and processing speed. Analysis of the subjects for group-time interactions revealed that the intervention group exhibited significantly improved executive function and brain function in the resting state. Additionally, gait speed and mobility were significantly improved at the follow-up. The VR-based training program improved cognitive and physical function in patients with MCI relative to controls (25).

A pilot study conducted in China in 2021 involving 18 patients with MCI (mean age 82.94 [SD5.44] years, 12 women) and 13 patients with mild dementia (mean age 85.7 [SD4.67] years, 10 women) found significant improvements in cognitive function after 5 weeks of VR-assisted cognitive training sessions. Both groups showed significant improvements in all cognitive function measurements [*p* < 0.001; (26)].

A meta-analysis by Kim et al. (27) on VR cognitive training for MCI or dementia found small-to-medium effect sizes of VR on outcome measures such as cognition (ES = 0.42, CI = 0.24, 0.60) and physical fitness (ES = 0.41, CI = 0.16, 0.65). However, the researchers found high variability in outcomes between the studies included in their analysis, and note that semi-immersive VR interventions had larger effect sizes than fully immersive interventions: “Perhaps VR-based intervention programs provide more diverse, comprehensive, and secure functions but can be complex and difficult for older adults to use” (pp. 7–8). Given that only two of the studies included used fully immersive VR, more research is needed into types of immersive VR that older adults find easy to use (27).

Similarly, a meta-analysis by Zhu et al. (26) based on the results of 11 high-quality RCTs showed that VR rehabilitation has positive effects on measures of cognitive and motor function such as attention, memory, visual–spatial-constructive function, and balance. A Primary analysis using a random effects model showed a significant moderately positive effect of VR on higher cortical functions (*g* = 0.45; 95% CI = 0.31–0.59; *p* < 0.001; *I*^2^ = 0%), attention and executive functions (*g* = 0.49; 95% CI = 0.26–0.72; *p* < 0.001; *I*^2^ = 31.45%), and memory (*g* = 0.57; 95% CI = 0.29–0.85; *p* < 0.001; *I*^2^ = 0%). Subgroup analysis showed that virtual reality sessions were more effective for patients with MCI (*g* = 0.46; 95% CI = 0.25–0.68; *p* < 0.0001) than for patients with dementia (*g* = 0.11; 95% CI = −0.47–0.70; *p* = 0.706). In contrast to Kim et al., the researchers found that fully immersive VR showed greater efficacy than semi-immersive VR, a finding they attribute to participants gaining a deeper experience of the VR environment (p. 11) (26).

Research has also found benefits of VR training for patients with cognitive deficits not associated with MCI; Chen et al. (29) in a meta-analysis of VR for poststroke cognitive impairment found improvement in such measures as Mini-Mental State Examination, Montreal Cognitive Assessment, and Loewenstein Occupational Therapy Cognitive Assessment (29).

Encouraging patients to perform VR- and game-based training may be beneficial to prevent cognitive decline. VR training programs can be more engaging than other forms of training, and thus improve adherence among target patient populations (30, 31). VR interventions may be an accessible, flexible, comprehensive, and cost-effective method of treating MCI and MCI-associated anxiety and depressive symptoms, especially for people who have difficulty attending outpatient appointments due to distance or mobility issues (31).

VR applications have potential to improve care of patients with MCI, including cognitive and behavioral therapy. However, almost all recent studies have been conducted in East Asian or Latin American populations; extant studies of VR cognitive training in other geographic and cultural contexts [e.g., (32)] are not recent enough to accurately reflect this rapidly changing technology. Due to the scarcity of experiments on fully immersive VR-based cognitive learning, further research is needed to establish its potential therapeutic efficacy (33).

Purpose of the study: To evaluate the effectiveness of a virtual reality-based program in addressing cognitive and affective disorders in patients with mild cognitive impairment during inpatient treatment.

## Materials and methods

The study included 62 patients undergoing treatment and examination in the Department of Geriatric Neurology of the Russian Gerontology Clinical Research Center, suffering from mild cognitive decline due to various organic diseases of the nervous system. The age of the patients ranged from 60 to 89 years.

Inclusion criteria were age 60 years or older, established diagnosis of mild cognitive impairment (MMSE ≥ 23 and ≤27) (34), and scheduled hospitalization in the Department of Geriatric Neurology of the Russian Gerontology Clinical Research Center.

Exclusion criteria were dementia, acute diseases or exacerbation of chronic diseases within 2 weeks before inclusion in the study, sensory deficits that could affect the ability to perform or evaluate study procedures, dermatitis or skin lesions on the face in the area where glasses are worn, and any other condition that, in the opinion of the investigator, could have interfered with a patient’s participation in the study.

Prior to this study, patients had received cardiovascular therapy (such as statins and ACE inhibitors), but had not received any neurological or antidementia therapy. Standardization of treatment was based on federal Clinical Guidelines for relevant nosologies adopted in the Russian Federation.

Participants over 60 years of age with MCI were prospectively recruited between May and December 2021. Among the 68 participants whose eligibility was assessed through structured clinical interviews, four withdrew voluntarily due to acute illness (*n* = 4) and unknown personal reasons (*n* = 2). Thus, the study included 62 patients with MCI undergoing treatment and evaluation (Table 1).

**Table 1:** Criteria for inclusion and exclusion of patients in the presented clinical trial.


Inclusion criteria
1. Men and women aged 60 years and older who are willing to participate in the study and have signed informed consent
2. Established diagnosis of pre-dementia cognitive impairment (MMSE ≥23 and ≤27)
3. Planned hospitalization in the neurology department of the Russian Scientific and Clinical Center
Exclusion criteria
1. Acute or exacerbated chronic illness within 2 weeks prior to study inclusion
2. Dementia
3. Sensory deficits that may interfere with the ability to perform or evaluate study procedures
4. Dermatitis or skin damage on the face in the area where glasses are worn
5. Any other condition that, in the investigator’s opinion, may interfere with the patient’s participation in the study

### Study design

The protocol for this study was approved by the ethics committee of Russian Gerontology Clinical Research Center (protocol №71 of April, 20, 2023).

This was an open-label randomized controlled trial designed to investigate the effectiveness of VR cognitive training in correcting cognitive and affective disorders in patients with mild cognitive impairment. Participants were randomly assigned to either the VR or control group. Unblinded restricted block randomization with 2 blocks consisting of half the sample size, with 1:1 distribution coefficient between blocks, was performed by drawing lots in the presence of the participants; The randomized pool of participants consisted of all eligible patients in the departments of undergoing study.

Prior to inclusion in the study, each patient’s medical history was carefully reviewed and each patient underwent detailed examination by a geriatrician and a neurologist. After screening, patients were randomized into treatment and control groups.

Both groups of patients received an identical rehabilitation program, which included three components:

1. Pharmacotherapy: choline alfoscerate at standard therapeutic doses for the entire observation period (2 weeks).
2. Cognitive training: daily sessions with a clinical psychologist/neuropsychologist, including standard paper-based cognitive exercises (memory, attention, arithmetic, and speech training) lasting approximately 30 min.
3. Measured physical activity: daily cardio exercise in the form of treadmill walking for 30 min under the supervision of a physical therapy instructor/rehabilitation therapist.

Thus, both groups experienced equal amounts of cognitive and physical stimulation, received equal attention from medical staff, and spent comparable amounts of time with therapists.

The treatment group also underwent a course of cognitive training with a VR exercise program for 2 weeks. Participants in the treatment group received VR cognitive training 5 times per week for a total of 10 sessions in addition to the standard treatment.

Before the start of therapy and at the end of the second week of the study, all patients were assessed for cognitive status using the MoCA scale, depression symptoms using the GDS-15 geriatric depression scale, and depression and anxiety using the HADS scale.

After successful completion of the prescreening, patients in the treatment group were sent universal VR headsets (Model: PICO G2 4k, Shenzhen, China). Any headsets handled by staff were disinfected, fabric surfaces cleaned with a disinfectant solution, and glass lenses disinfected with an alcohol-based lens cleaner followed by a 1-min ultraviolet cleaning.

Virtual reality training lasted a total of 20 min for a total of five times per week during the 2 weeks of the study. All interventions included five modules with defined daily training sequences. Participants were given the option to stop the intervention at any time, but all completed the planned treatment.

The Virry VR program used in the study was developed by Fountain Digital Labs (London). It includes relaxation exercises and interactive games developed using evidence-based principles of cognitive behavioral therapy (CBT), mindfulness meditation, and physiological biofeedback therapy using embedded biometric sensors. The program provides a combination of skills training and treatment related to CBT through scheduled daily virtual sessions. Each VR session lasts from 3 to 6 min (interactive) and 5 to 25 min (meditation), and utilized such software components for Virry, as “Virry Happy” and “Virry Drozdov.” The virtual landscapes used in the Virry program are based on the African savannah, using footage from the Lewa wildlife conservancy in Kenya, and include animals and natural features such as lakes, intended to calm the patient and awaken their interest in life and nature. In a typical interactive session, a participant might feed, observe, and learn about animals such as elephants, lions, and vervet monkeys. In a typical meditation session, a patient might be prompted to concentrate on their breathing and relax contemplating the savannah.

During the VR therapy, any adverse events were recorded.

After completing the course of therapy, all patients underwent repeated cognitive testing, and subjective wellbeing data was collected in the form of simple verbal descriptions, such as: changes in emotional wellbeing, the degree of anxiety and the presence of current experiences, feelings of fatigue, etc.

Statistical processing of the results was performed using STATISTICA 10.0 statistical program with packages. Normality of distribution was assessed; all parameters were not normally distributed. The results are presented as M ± SD and as Me (Q1: Q3). Significance of changes in the values of scales during 2 weeks of observation was revealed using the paired Wilcoxon criterion, and differences between the control and experimental groups in terms of changes in test results were calculated using the Mann–Whitney criterion. Differences were considered significant at *p* < 0.05.

## Results

The results of laboratory tests of all patients were within normal limits, including general and biochemical blood tests. The study included 62 patients who underwent treatment for 2 weeks. No patients were excluded during study. Characteristics of patients participating in the study are presented in Table 2.

**Table 2:** Participant characteristics.

Characteristics	Treatment group (*n* = 31 patients)	Control group (*n* = 31 patients)
Average age	72.8	69.2
Men	4 (12.9%)	9 (29.0%)
Women	27 (87.1%)	22 (71.0%)

Patients in the two groups did not statistically differ in age, duration of disease, or presence of such risk factors as atherosclerosis, coronary heart disease, hypertension, diabetes, craniocerebral trauma in the anamnesis, or family history of dementia. All patients received recommendations on healthy lifestyle and nondrug therapy (smoking cessation, physical activity, hypocholesterolemic diet). Patients in both groups at the time of inclusion in the study were comparable in terms of severity of cognitive impairment (22.5 ± 2.2 and 22.9 ± 2.5 points) on the MoCA scale and symptoms of depression and anxiety (on the GDS-15 and HADS scales) in the treatment and control groups, respectively (see Table 3).

**Table 3:** Dynamics of neuropsychological testing indicators before and after treatment.

Scale	Treatment group (*n* = 31 patients)			Control group (*n* = 31 patients)		
	Before treatment (M; Me)	After treatment (M; Me)	*р*	Before treatment (M; Me)	After treatment (M; Me)	*р*
MoCA	22.5 ± 2.2; 23 (21:24)	25.1 ± 2.3; 26 (24:26.5)	<0.0001	22.9 ± 2.5; 23 (21:25)	24.2 ± 2.7; 24,5 (22:26)	**0.001**
GDS 15	5.3 ± 3.3; 4.5 (2.75:7)	4.0 ± 3.2; 4 (1.75:5.25)	0.014	5.8 ± 3.1; 6 (3.75:8)	4.9 ± 2.9; 5 (3:7)	**0.007**
HADS depression	5.9 ± 3,2; 5 (4:8)	4.3 ± 3.2; 4 (2:7)	0.007	5.6 ± 3.1; 6 (3.75:8)	5.4 ± 3.1; 6 (3:7)	**0.327**
HADS anxiety	7.7 ± 3.5; 7 (4.75:10)	6.3 ± 3.6; 7 (3:9)	0.014	7.6 ± 3.2 6 (5:9.25)	6.9 ± 3.4 7 (4.5:9)	**0.793**

The differences are statisticaly significant at the *p* < 0.001 level.

During the study, no serious complications preventing the continuation of virtual reality activities or any other exclusion criteria were observed. Subjectively, the patients in both groups noted improvement in their subjective condition and increased ability to concentrate. Both the standard and experimental treatments were well tolerated. No adverse events were identified.

Table 3 shows results for both groups before and after treatment on the MoCA scale (cognitive ability), GDS-15 (depression), and HADS (depression and anxiety).

Both groups showed statistically significant improvement in cognitive function on the MoCA scale and reduction of depressive symptoms on the GDS-15 scale after 2 weeks of treatment (control group, *p* = 0.001; treatment group, *p* < 0.0001). In the group of patients who underwent training using VR, statistically significant improvement in the anxiety and depression subscale of the HADS scale was also observed (*p* = 0.007; Table 4). Patients in the treatment group showed significantly higher improvement in cognitive function scores on the MoCA scale (*p* = 0.007), and in depressive symptoms on the HADS depression subscale (*p* = 0.05). The difference in the changes in the test scores of the main group compared to the control group are shown in Table 4. Differences between GDS and HADS anxiety scores were not statistically significant.

**Table 4:** Changes in neuropsychological testing indicators in treatment and control groups.

Scale	Change over 2 weeks: mean ± standard deviation (min; max)		*р*
	Treatment group (*n* = 31 patients)	Control group (*n* = 31 patients)	
MoCA	2.6 ± 2.1 (0.8)	1.3 ± 1.5 (−1.5)	**0.007**
GDS	−1.3 ± 2.6 (−10.3)	−0.9 ± 1.7 (−6.3)	**0.937**
HADS depression	−1.6 ± 3.2 (−11.4)	−0.3 ± 1.5 (−4.3)	**0.050**
HADS anxiety	−1.4 ± 2.9 (−10.5)	−0.7 ± 2.0 (−5.3)	**0.430**

The differences are statisticaly significant at the *p* < 0.001 level.

## Discussion

Our study on patients with MCI showed that training with VR technologies compared to standard treatment leads to more significant improvement in cognitive function as measured by the MoCA scale, a result generally consistent with international studies (6, 26, 27, 29, 33–35). Our study also demonstrated a positive effect of using VR programs on depression symptoms in patients with MCI, which is consistent with the findings of a systematic review by Chan et al. (18) demonstrating that cognitive training programs using virtual reality improve cognitive function and emotional status in patients with MCI.

Treatment of mild cognitive impairment has two main goals:

- Secondary prevention of dementia, slowing the progression of cognitive impairment;
- Reducing the severity of existing impairments to improve the quality of life of patients and their families.

One promising strategy is comprehensive, non-pharmacological prevention of cognitive decline. The results of the multicenter, randomized, controlled FINGER trial, conducted in Finland, assessed the effectiveness of non-pharmacological prevention methods, such as nutritional optimization, exercise, cognitive training, and social activity. The study included 1,260 patients (men and women) aged 60 to 77 years at increased risk of developing dementia. Patients were randomly assigned to two groups of approximately equal size. The study group employed the non-drug preventive measures described above over a two-year period, including individual and group consultations with a nutritionist, regular strength and aerobic exercise supervised by instructors, computerized cognitive training sessions, and social activities. Cardiovascular and metabolic risk factors were also monitored. Patients in the control group received regular healthy lifestyle advice during this period. The primary endpoint of the study was an assessment of cognitive function using extensive neuropsychological testing. Results showed that these methods significantly slowed the progression of attention and executive function disorders, but had little effect on memory. This is likely due to the fact that these methods significantly slow the progression of cerebrovascular injury and have a lesser effect on the rate of neurodegenerative processes. Thus, an individually developed management plan for patients with MCI can often reduce the severity of existing impairments and prevent or delay the development of dementia (36, 37).

Previous literature has shown conflicting results regarding the effectiveness of fully immersive VR compared to less immersive types. The results of this study support the effectiveness of immersive VR due to the device used, while other types were not tested. While Kim et al. (27) note that older adults may find fully immersive VR challenging to use, participants found the Virry VR system enjoyable to use and did not report significant challenges.

Given the consistency of findings showing that VR cognitive training is effective for MCI, it is important to assess which forms of cognitive training are more effective. A meta-analysis by Moulaei et al. (38) found that VR-based games are more effective than VR-based education programs. Our study contributes to understanding this topic by showing a VR program based on education and meditation/relaxation in a nature setting to be both effective and readily accepted by study subjects.

Our study showed no significant improvement in the treatment compared to the control group on the other scales used, GDS and HADS Anxiety. Existing research suggests that the GDS has limited validity in cases of cognitive impairment (39, 40); this is a possible reason for the disparity in results for the two depression scales in the present study.

This discrepancy can be explained by several factors:

1. Sensitivity to short-term changes: The GDS-15 is designed to assess more stable, trait-like depressive symptoms in the elderly, excluding somatic complaints. A two-week course of VR therapy likely has a more pronounced effect on acute, situational affective reactions and general emotional tone (state-like), which are better captured by the HADS.
2. Overlap with anxiety: The HADS depression subscale contains elements that overlap with anxiety symptoms (e.g., psychomotor restlessness). Because the VR environment (the Virry program) provides rich multisensory stimulation and elements of relaxation, it may have more quickly alleviated this mixed anxiety-depressive component, which was reflected in HADS scores, but not the GDS.
3. Floor effect: Baseline GDS-15 scores were relatively low (5.3 and 5.8), consistent with mild depression. With such baseline values, the scope for further significant between-group differences over a short period (2 weeks) is limited.
4. Study power: The sample size of 31 patients per group may be insufficient to detect small but clinically significant between-group differences in anxiety scores.

Given previous research showing immersive VR training to be effective at reducing anxiety in elderly subjects (41), the reason for our finding of no significant difference between treatment and control groups on the HADS Anxiety scale is unknown; a longer course of treatment might be needed.

It is possible that the relatively short 1-month training period might have resulted in the lack of group difference, as a learning effect may have impacted the posttraining neuropsychological test results in the control group. However, the improvement in visuospatial function in the VR group, even after the short period of cognitive training, might be attributed to the ecological nature of the fully immersive VR environment. In the enriched auditorily and visually stimulating environment, processing of visual orientation, visuospatial construction, and visual selective attention likely occurred (42, 43). In recent studies with VR evaluation, investigators have been able to effectively differentiate between the navigational and visuospatial deficits observed in patients with MCI and healthy older people (44, 45). We believe that the cognitive training performed in the maximally immersive environment with the virry in our study might have increased visuospatial functioning in those at a predementia stage. The immersion methods utilized in previous studies investigating VR cognitive training in older people have employed desktop-based systems, screen and sensors (20), screen and glasses, and head-mounted display and fixed joystick setups. Although heterogeneity in study populations and methodological differences among prior studies have resulted in inconsistent findings, this study provides further evidence to support the benefits of VR cognitive training in eliciting improvements in visuospatial processing through the repeated presentation of real-world, dynamic, multisensory, and interactive environments (20, 22).

As noted previously, the study presented has several important limitations, including low sample power, lack of blinding or substitute intervention, and a lack of more detailed assessment of the functional status of the subjects. However, the results obtained appear to be clinically significant for a low-cost adjunctive therapy, comparable in potency to other standard treatments. In pre-dementia cognitive impairment, even a small additional benefit can be critical to maintaining a patient within the normal range or mild impairment, delaying the transition to dementia. From an economic and clinical perspective, if VR training (especially when used with affordable standalone headsets) can enhance the effects of standard therapy and potentially delay the need for more expensive care or institutionalization, these costs and time may be justified.

As a conclusion, VR cognitive training technologies might be a promising method of additional therapy that could lead to improved quality of care for patients with mild cognitive impairment.

Further research is needed to develop the most simple, convenient, and effective programs using virtual reality for cognitive training for patients with different types of MCI, including study improvement in different ways to overcome existing limitations.

## Data Availability

The original contributions presented in the study are included in the article/supplementary material, further inquiries can be directed to the corresponding author.
